# Analysis on alteration of road traffic casualties in western China from multi-department data in recent decade

**DOI:** 10.3389/fpubh.2022.972948

**Published:** 2022-11-10

**Authors:** Jinlong Qiu, Guodong Liu, Ao Yang, Kui Li, Hui Zhao, Mingxin Qin

**Affiliations:** ^1^Institute for Traffic Medicine, Daping Hospital, Army Medical University, Chongqing, China; ^2^College of Biomedical Engineering, Army Medical University, Chongqing, China

**Keywords:** road traffic safety, accident analysis, accident severity, accident characteristics, Western China

## Abstract

**Background:**

Road traffic safety has considerably improved in China. However, the changes may differ in the economically backward and altitude higher western region. This study aims to investigate changes in the occurrence and severity of traffic casualties in western China and illuminate several key causal factors.

**Materials and methods:**

Traffic accident data from the Annual Traffic Accident Statistics Report combined with population and vehicle data from the China Statistics Bureau between 2009 and 2019, were retrospectively analyzed. Traffic accident numbers, fatalities, human injury (HI), case fatality rates (CFR), mortality per 100,000 population (MRP), and mortality per 10,000 vehicles (MRV) were compared between the western and eastern regions. The HI, CFR, MRV, and MRP between the four groups based on the altitude of cities, below 500 meters, 500 to 1,500 meters, 1,500 to 3,000 meters, and over 3,000 meters, were compared using one-way analysis of variance. One hundred and seventy-eight cases of extremely serious traffic accidents were further analyzed in terms of accident occurrence time, vehicle type, road grade, road shape, accident pattern, and accident reason. The differences of accident characteristics between the eastern and western regions were compared using the chi-square test.

**Results:**

The number of traffic accidents and fatalities decreased in low-altitude areas in western China. However, there was a significant increasing trend in the high altitude area. The HI, CFR, MRV, and MRP were higher in the western region than that in the eastern and national. Those accident indicators tended to increase with increasing altitude. And there were statistically significant differences (*p* < 0.05) among groups from different altitudes. Chi-square test results show that there are statistically significant differences (*p* < 0.05) in term of road grade, road shape, accident pattern between eastern and western. Low-grade roads, combined curved and sloping roads, and rollover were significant features associated with traffic accidents in the western region. Bad roads were the main cause of rollover accidents in western China, which will lead to more serious casualties. Over speeding, overloading, bad weather, vehicle failure, and driver error were the top five accident causes.

**Conclusion:**

Traffic accidents are increasing in high-altitude areas of western China, and they lead to more severe casualties. The characteristics of serious traffic accidents in this part of the country differ from those of the eastern regions. Improving road safety facilities, restrictions of speed, and improving medical treatment at accident scenes may be effective measures to reduce traffic accidents related injuries in the western region.

## Introduction

Globally, road traffic accidents have become a major public health concern, which caused more than 1.35 million deaths and 50 million injuries every year (1). Road traffic injuries have become one of the leading contributors to regional and global disease and injury burden, especially in low- and middle-income countries (2, 3). The increase in the number of accidents has been curbed, but there are still a large number of casualties (4). The regional distribution of road traffic accidents in China, similar to the global situation, varies significantly. In addition, registered drivers and vehicles are increasing rapidly in the western region owing to the development of the Chinese economy. Road traffic condition in this region may therefore be changing (5). Therefore, road traffic accident prevention in western China needs more attention.

The most typical geographical feature of western China was the high altitude (6). There were numerous risk factors that contribute to traffic accidents in high altitude areas, including longer steep road sections, snow blindness among drivers due to mountain snow, and fatigue during driving that occurs due to low partial pressures of oxygen (7, 8). On the other hand, trauma injuries were more severe in high-altitude regions because of the high incidence of shock, reduced resuscitation capacity tolerance, and early occurrence of multi-organ failure (9). Furthermore, the western region accounts for 72% of the total land area. However, owing to considerably disproportionate development in the different regions, the western region contributes to only 20.1% of the gross domestic product (10). Building roads at high altitudes remains a challenge due to the lack of road construction investment. In addition, roads at high altitudes are usually considered to be bad due to topographical features. The bad roads and limited medical resources may have prevented the injured from receiving timely treatment and possibly caused serious casualties (11). Based on the above reasons, the population in the West might suffer from more serious public health threats (12, 13).

The number of accidents and fatalities is a direct indicator of the basic traffic safety situation. Commonly used additional evaluation indices include the quantity per unit of population, quantity per unit of registered vehicles (14). These indices have been used for time series analysis or cross-country comparisons and to estimate the overall level of traffic safety on the road. However, there were little literature on accident severity and its variation over time, as the population fatality index is an indicator that combines population accidents and accident fatalities. Hayakawa et al. (15) introduced two indicators, namely, human injury (HI) and case fatality rate (CFR), to compare traffic accident risks in Japan and the United States and confirmed their potential for explaining cross-national differences in risk perceptions. A study on changes in traffic accident severity in China also used similar indicators (16). Although numerous studies about road traffic crashes have been conducted worldwide, few have focused on altitude-related differences in road traffic injuries.

How and why traffic accidents happen is another concern of traffic safety, which is important for accident prevention. Factors related to traffic safety, including drivers, vehicles, roads, and environmental element have been studied (17). Driving is a complex cognitive-behavioral process of feeling, analyzing, judging, and responding to the external environment (18). The human factor, such as hazardous or unsafe behaviors, play a crucial role in road traffic accidents. Evidence indicates that 70–90% of road accidents are caused by road user behaviors, including overspeed, drinking drive, Fatigue, and illegal passing (19, 20). Analysis of risk factors in traffic accidents is an effective way to develop accident prevention measures. Considerable efforts have been made to analyze accident times, seasons of occurrence, weather and road conditions, accident patterns, and accident risks using ordinary statistical methods and various models including artificial neural networks, spatial and temporal correlations, and multinomial logistic models (21–24). A preliminary study on alterations in road traffic accidents in China between 2006 and 2013 were conducted by our research team (5). However, only the number of accidents, fatalities, and injuries were considered in this study. There is a lack of in-depth discussion on accident severity and risk factors, especially among cities of different altitude.

China is one of the countries with the highest numbers of traffic injuries worldwide. In-depth investigation and analysis of traffic accidents based on historical data offer an effective method for developing effective prevention strategies. An exemplary accident investigation and registration system has been established in China. The Road Accident Statistical Annual Report, issued by the China Traffic Management Bureau of the Public Security Ministry, is an invaluable data source. The annual report records the number of traffic accidents, fatalities, and injuries by province, from which national trends in road safety can be analyzed. The national accident data does not include detailed information on individual accidents. While, typical extreme serious traffic accidents containing detail information on human, vehicles, roads, and environment, which would better reflect the characteristics of traffic accidents, were also record in the annual report.

Due to the high altitude and economically backward in western China, it is hypothesized that traffic accidents in the western region may have different characteristics from those in the eastern region. Road traffic situation of western China should be further analyzed and develop targeted prevention policies. The present study has two main objectives. The first purpose was to compare the changes of occurrence and severity of road traffic accidents from the aspect of western and eastern of China and cities in different altitude. The second purpose was to analysis the difference of accident characteristics and human risk factor between western and eastern and propose some countermeasures.

## Materials and methods

### Data source

The data on accidents for this paper were mainly obtained from the Road Accident Statistical Annual Report, published by the Traffic Management Bureau of the Public Security Ministry of China (25). Regional data, including population, city altitude, and vehicle ownership were obtained from the provincial department of statistics or the National Bureau of Statistics (26–32). Ethical approval was obtained from the ethics committee of Army Medical University (2022–214).

The accidents were divided into two categories, namely, general accidents and extremely serious accidents. A general accident was one that caused one or two serious injuries, or three or more minor injuries. The extremely serious traffic accident referred in this research was defined as a single accident that resulted in more than 10 deaths. The general accidents data was used to investigate the accident incidence and severity of traffic casualties. The accident characteristics and possible reasons were analyzed using the extremely serious traffic accident, which have the more detailed information.

The entire accidents occurred were enrolled to analysis the change of occurrence and severity of fatality in western China. Data of 91 cities in western China, where regional data such as altitude, population, and vehicle data could be obtained, were used to analyze the differences between cities at different altitudes. Extremely serious accidents were entire national data, which will be used to determine the typical characteristics and causes of accidents in the western region. The relevant data collected between 2009 and 2019.

### Variable definition and classification

#### Indicators of accident incidence and severity

Accidents were analyzed based on the six following indicators: the number of accidents and fatalities, MRP, MRV, HI, and CFR. They are defined as follows:

(1) $$\begin{array}{l} \text{MRP}=\text{F}/\text{P}\times 100,000 \end{array}$$

(2) $$\begin{array}{l} \text{MRV}=\text{F}/\text{V}\times 100,00 \end{array}$$

(3) $$\begin{array}{l} \text{HI}=\left(\text{F}+\text{I}\right)/\text{A} \end{array}$$

(4) $$\begin{array}{l} \text{CFR}=\text{F}/\left(\text{F}+\text{I}\right) \end{array}$$

In Equations, F denotes the total fatalities, I indicates total injuries, P denotes the populations, V denotes the vehicle ownerships, and A denotes total accidents. Notably, minor accidents such as scratches were ignored. A similar calculation was used by Hayakawa et al. (15) and Wang et al. (16) to evaluate the lethality of accidents.

#### Accident characteristics and risk factors

Extremely serious accident records included the date, time, location, deaths and casualties, vehicle type, quantity and categories of accident type, main causes, and a detailed description of the crash. The accident causes included human, vehicle, road, and environmental factors. Human factors included those that limited the ability to drive or promoted risk-taking behavior on a short- or long-term basis. These included tailgating, fatigue, and habitual speeding, among others. Vehicle factors included all factors related to vehicle safety status, such as vehicle breakdown, overload status, and insurance status, among others. Road factors referred to the road grade and shape. Environmental factors referred to the influence of time and space, these included the time of the accident, light conditions, visibility, and weather, among others. In this study, in order to better analyze the accident characteristics, accident variables were redivided into six categories: accident time, vehicle type, road grade, road shape, accident pattern, and accident reason. The classification of factors, data collection, and description of variables were shown in Table 1.

**Table 1:** The classification of factors, data collection, and description of variables.

**Variable**		**Description**	**Data collection**	**Factors**
Accident time		Two level (Day and Night) 6 AM-18 PM is day, 18 PM to 6 AM is night	Record by police	Environment factors
Vehicle type		Three level (Small passenger car, Medium/ Large passenger car, and Truck)	Type on vehicle license, photos taken by police on the scene	Vehicle factors
Road grade		Six level (Highway, Level 1, Level 2, Level 3, Level 4, and Other)	Record by police	Road factors
Road shape		Five level (Flat straight, curve, slope, combined curve and slope)	Record by police	Road factors
Accident pattern		Six level (Rollover, frontal, rear-end, fire and other, side, fixed object)	Photos taken by police on the scene	
Accident reason	Over speed	Yes or No, Exceed the road speed limit,	Speed camera, calculated by expert	Hunman factors
	Overload	Yes or No, cargo or occupants exceeds the limit	Weight/passenger limit on license, weight record through transport company and toll station	Vehicle factors
	Bad weather	Yes or No, Weather record of Meteoro-logical Bureau, real time record on the scene	Weather record of Meteoro-logical Bureau, real time record on the scene	Environment factors
	Vehicle failure	Yes or No, Brake problem and others	Vehicle examination report	Vehicle factors
	Driver error	Yes or No, Not illegal, but improperly operated	Accident analysis report	Hunman factors
	Drive opposite	Yes or No, Occupy the opposite lane	Accident analysis report	Hunman factors
	Illegal drive	Yes or No, driving without license, or driving under influence (alcohol or grug)	Driving license, record by police	Hunman factors
	Fatigue	Yes or No, the start and rest time of trip	Regulations for the implementation of road traffic safety law	Hunman factors
	Illegal passing	Yes or No, Passing in inappropriate places	Accident analysis report	Hunman factors
	Tailgating	Yes or No, Fail to maintain a safe distance from the vehicle in front	Accident analysis report	Hunman factors

### Statistical analysis

Data were inputted using Microsoft Excel (Microsoft Excel for windows version 16.14.1). After collection, the data were checked for completeness, cleaned, and edited by removing missing values. For example, if the population data was missing for a certain year, the fatalities in the corresponding year were excluded during MRP calculation. The data were tested for normality before analysis of variance. One-way analysis of variance (one-way ANOVA) was used to compare the mean differences between groups in terms of altitude. The chi-square test was used to assess significant differences between the western and eastern groups, with *p* < 0.05 being considered statistically significant. The statistical analysis was performed using the SPSS, (SPSS Statistics Version 23, Armonk, NY, USA).

## Results

### Incidence of traffic accidents

**Figure 1:**
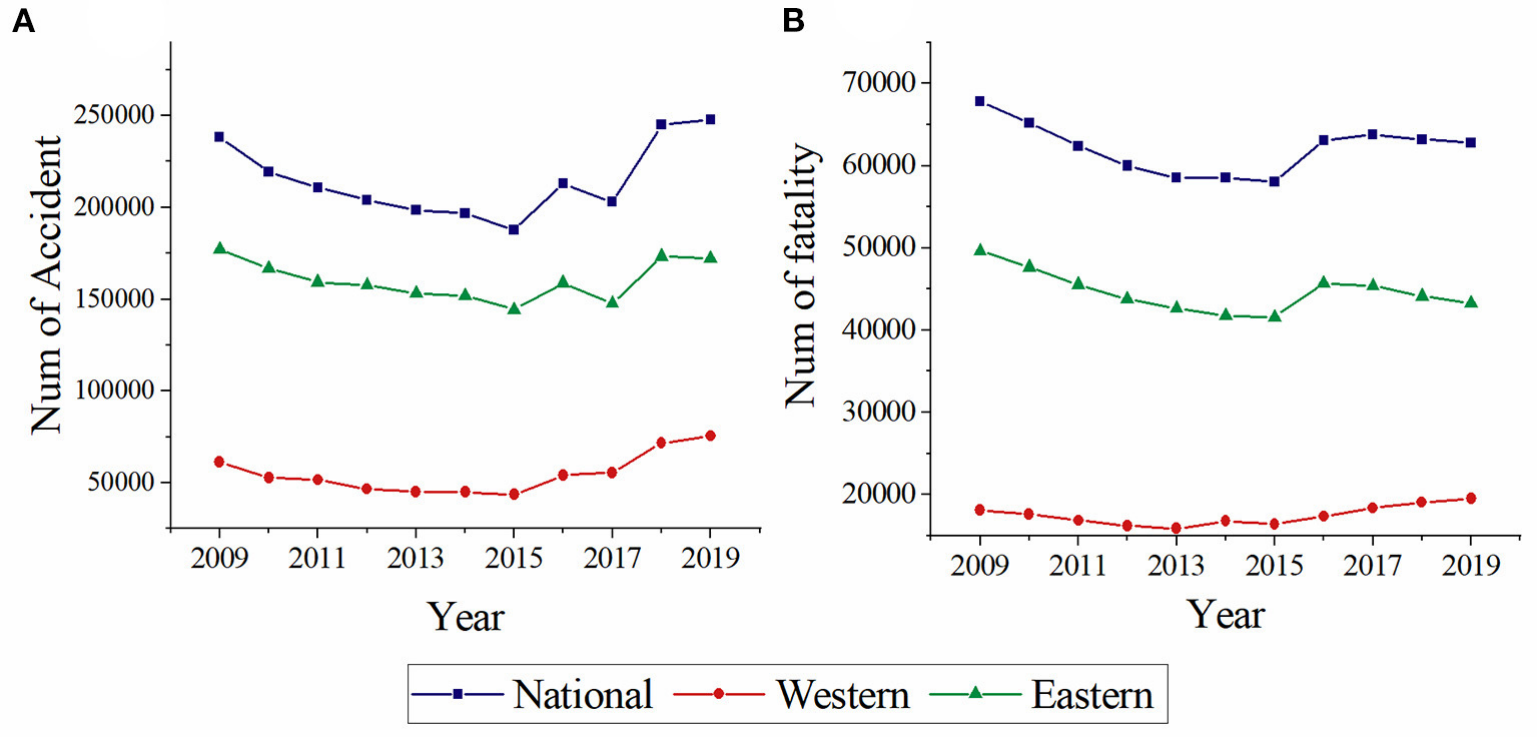
Annual distribution of traffic accidents **(A)** and fatalities **(B)** at western, eastern, and national.

The number of traffic accidents and fatalities during the decade were shown in Figure 1, with the number decreased annually from 2009 to 2015. While, an increasing trend has emerged after 2015. The accidents and fatalities in western increased by 73.55 and 18.65%, respectively. The values in Eastern are 19.31 and 4.03%.

**Figure 2:**
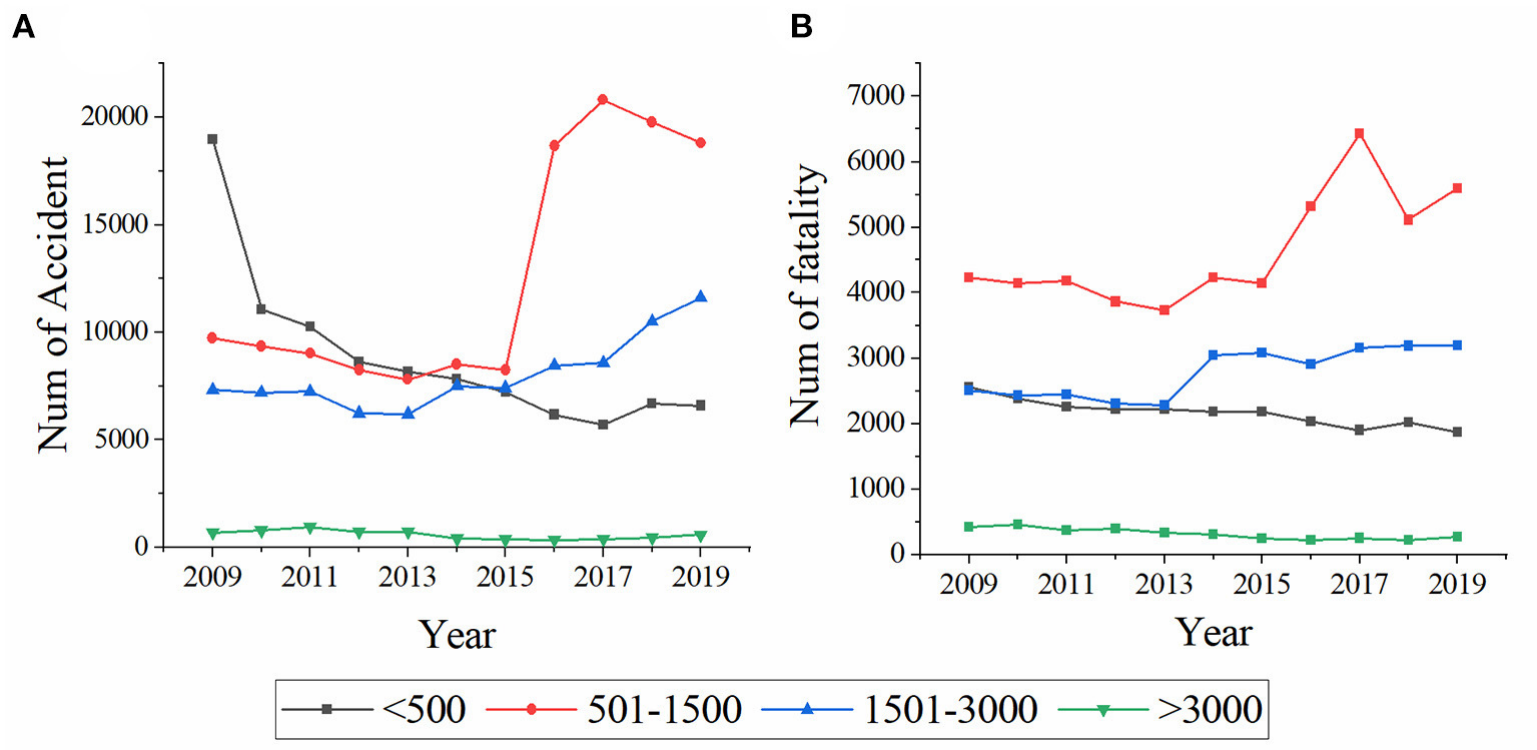
Annual distribution of traffic accidents **(A)** and fatalities **(B)** at different altitudes.

The number of traffic accidents and fatalities that occurred in the four altitude groups were shown in Figure 2. The average numbers of accidents per group were 8,848, 12,637, 8,092, and 586, respectively. In the below 500 meters group, the accident number declined significantly over the past decade. In contrast, there was an overall upward trend for the 500 to 1,500 meters and 1,500 to 3,000 meters groups. The average fatalities per group were 2,165, 4,634, 2,801, and 316, respectively. The trend of fatalities over time was similar to that of the number of traffic accidents.

### Severity of accident casualties

**Figure 3:**
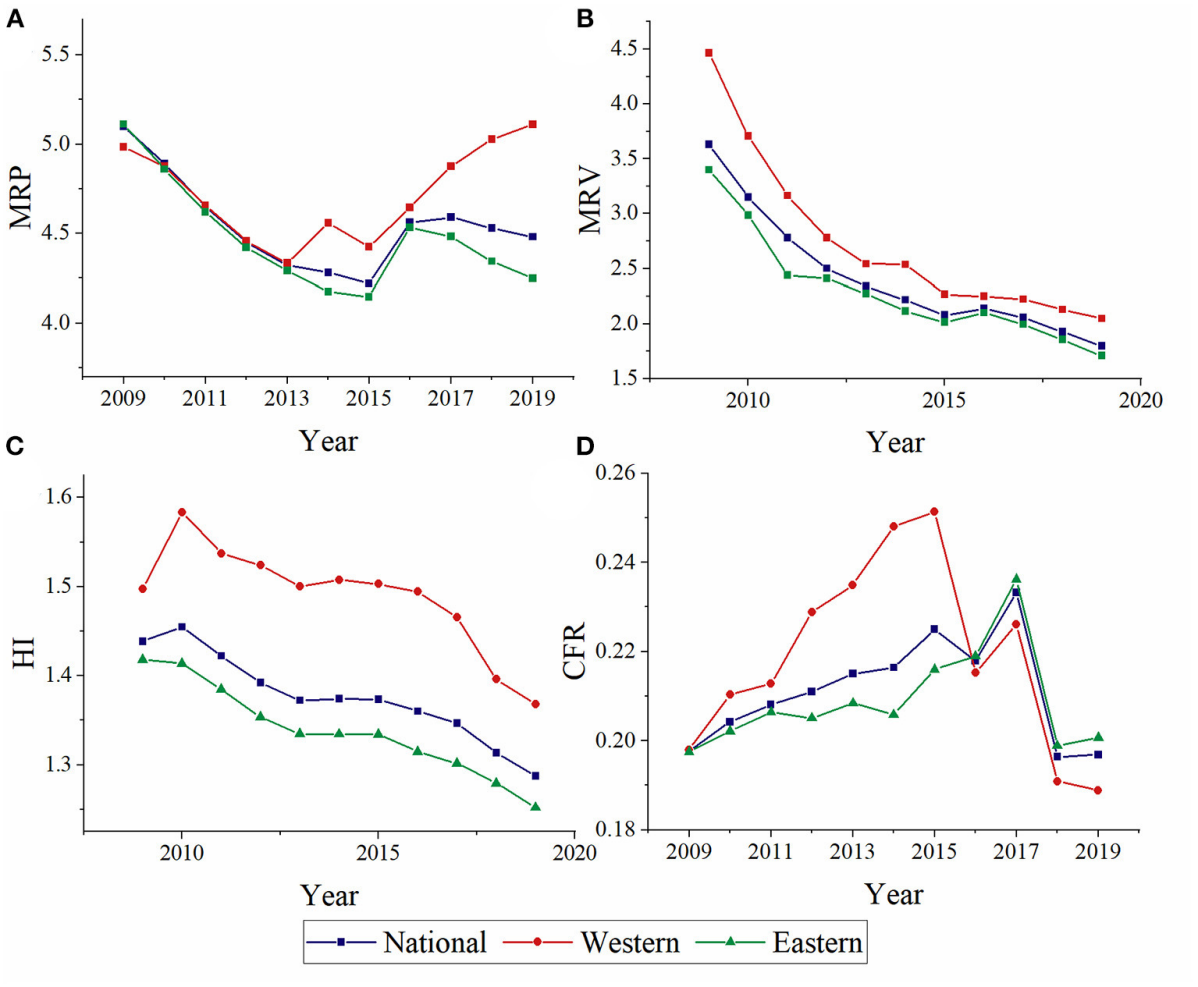
Annual distribution of MRP **(A)**, MRV **(B)**, HI **(C)**, and CFR **(D)** at western, eastern, and national.

The comparison of MRP, MRV, HI, and CFR between eastern and western were shown in Figure 3. The HI and MRV present a decreasing trend. The MRP shows a downward and then upward trend. On the contrary, CFR shows a downward and then upward trend. These four indicators were almost highest in western China, except the CFR from 2016 to 2019.

In order to compare the differences in accident severity between high and low altitude areas, the HI, CFR, MRP, and MRV were analyzed using one-way ANOVA. The results were shown in Table 2.

**Table 2:** One-way ANOVA results.

**Indicator**	**Average**				**One-way ANOVA**	***P*-value**
	**<500**	**500–1,500**	**1,501–3,000**	**>3,000**		
HI	1.406	1.596	1.539	1.763	25.870	0.000^*^
CFR	0.189	0.251	0.228	0.318	119.402	0.000^*^
MRP	2.951	5.142	4.844	6.713	13.676	0.000^*^
MRV	1.883	2.892	2.507	3.364	12.946	0.000^*^

* Refer to the differences are statistical significance with *p* < 0.05.

**Figure 4:**
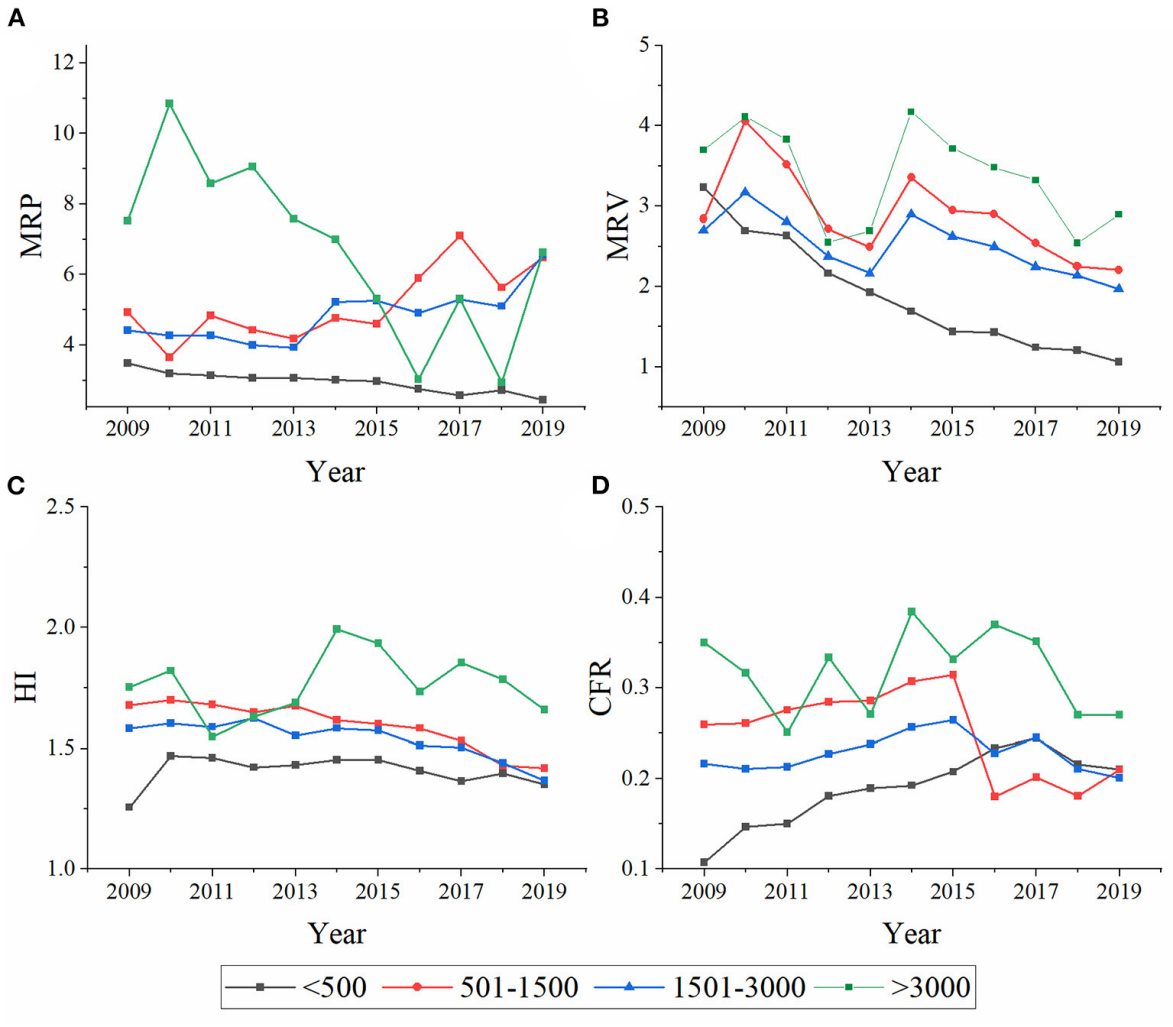
Annual distribution of MRP **(A)**, MRV **(B)**, HI **(C)**, and CFR **(D)** at different altitudes.

In terms of geographical distribution, the HI in the above 3,000 meters group was significantly higher than that in other regions. The HI gradually decreased with a decrease in altitude (Figure 4A). Statistically significant differences were found among the groups (*p* = 0.000).

In terms of time distribution, the CFR showed a trend of gradual increase before 2015 and gradual decrease after that point. There were statistically significant differences between the groups (*p* = 0.000). The CFR in the above 3,000 meters group was significantly higher than that in the below 500 meters group (Figure 4B).

From 2009, a general decrease in the MRP was observed in the above 3,000 meters and below 500 meters groups. However, the other two groups showed a slightly increased trend (Figure 4C). The differences among the groups were statistically significant (*p* = 0.000). Except for a relatively dramatic increase in 2014, the MRV generally showed a downward trend (Figure 4D). Statistically significant differences were observed among the four groups in terms of the average MRV (*p* = 0.000).

### Accident time and vehicle type

Overall, among 178 vehicle accidents that occurred during the day, 76 (67.5%) and 50 (75.8%) occurred in the eastern and western regions, respectively. There was no significant statistical difference between the western and eastern regions in terms of accident time (*p* = 0.860). Passenger cars or trucks accounted for the highest proportion of accident vehicle types, with 58% in eastern and 59.1% in western regions. Similarly, there was no statistical difference between the western and eastern regions in terms of vehicle type (*p* = 0.110).

### Accident roads

**Figure 5:**
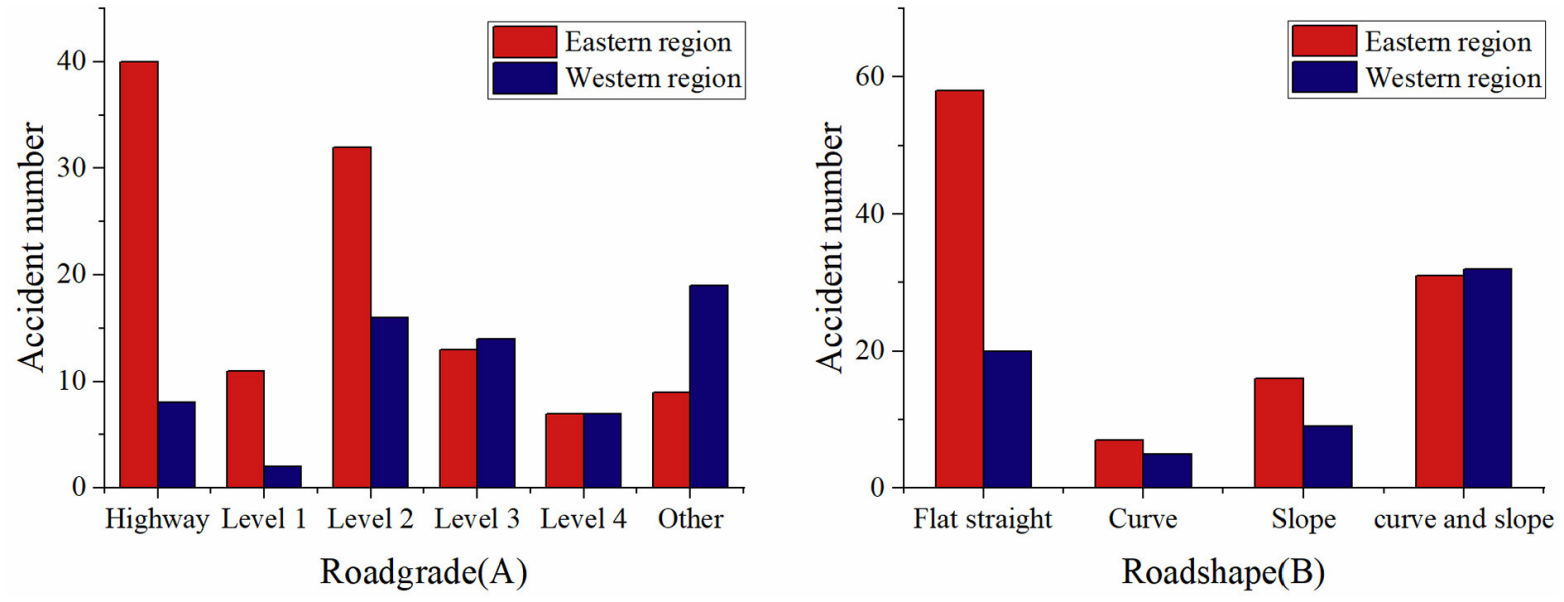
Distribution of road grades **(A)** and road shapes **(B)** for extremely serious traffic accidents in the eastern and western regions.

According to the classification of roads by the administrative department, roads in China were categorized into expressways, level 1–4 roads, and other roads. In the eastern region, more than one-third of cases were occurred in highways (35.7%), followed by level 2 roads (28.6%). In the western region, below level 2 roads accounted for 84.9% of cases, followed by other (28.8%), level 2 (24.2%), level 3 (21.2%), and level 4 (10.6%). A statistically significant difference was observed between the western and eastern regions in terms of road grade (*p*=0.000). Distribution of road grades were seen in Figure 5A.

The road shape was classified as flat and straight, curved, sloped, and combined curved and sloped. In the eastern region, more than half of all accidents occurred on flat straight roads (51.8%). In the western region, most accidents occurred in combined curved and sloped (48.5%). A statistically significant difference was observed between the western and eastern regions in terms of road shape (*p* = 0.023). Distribution of road shape were seen in Figure 5B.

### Accident patterns

**Figure 6:**
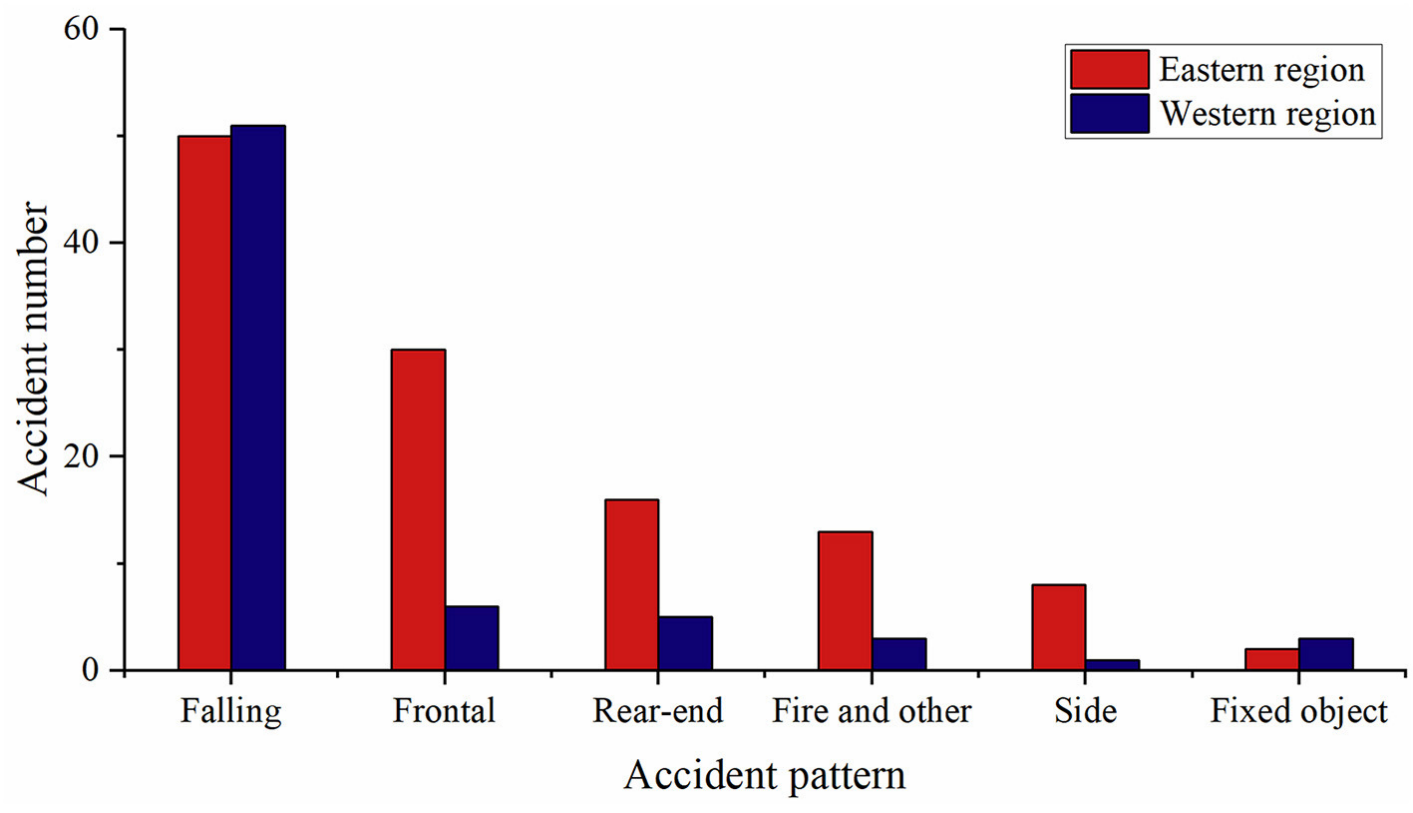
Extremely serious traffic accident patterns in the eastern and western regions.

The extremely serious traffic accident patterns were illustrated in Figure 6. Among all crashes, vehicle falling from a height (including rolling) was the most common pattern. In the western region, the percentage of fall-related accidents reached 73.9%. Statistically significant differences were found between the western and eastern regions in terms of accident pattern (*p* = 0.001).

### Causes of accident

**Figure 7:**
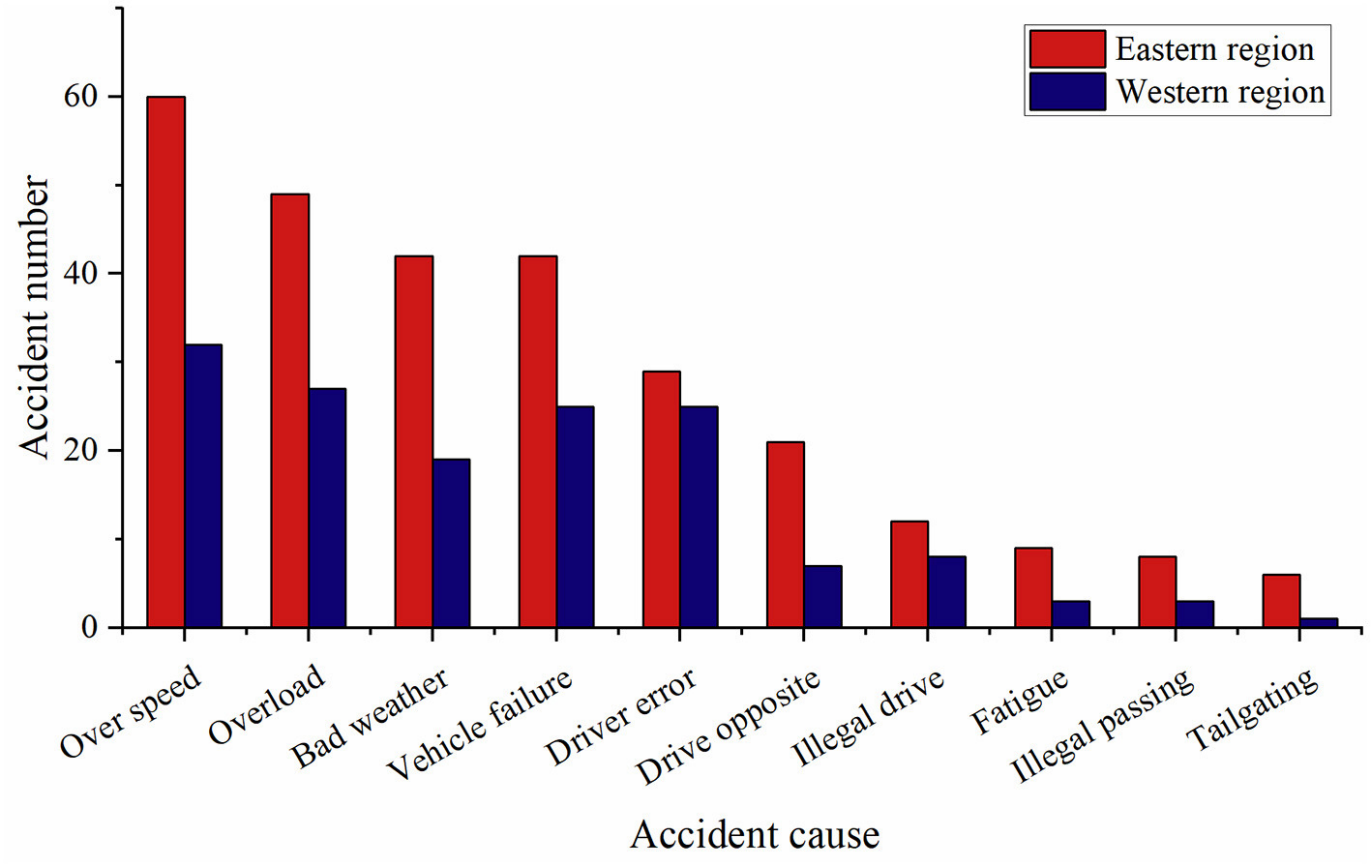
Distribution of reasons for extremely serious traffic accidents in the eastern and western regions.

Among the crashes, over speeding, overloading, bad weather, vehicle failure, and driver error were the top five causes as presented in Figure 7. However, a statistically significant difference was not found between the western and eastern regions in terms of accident reason (*p* = 0.619).

## Discussion

Great change has occurred in road traffic conditions of China. Our results showed that the number of accidents and fatalities in China continued to decrease from 2009 to 2015, which was consistent with the finding of another research team in our country (33). The reasons might be due to the increased investment in national health and transportation construction, the overspeed limits on driving, and the application of seat belt, which all have an important impact on reducing road accident (34, 35). However, the frequency of road accident both in western and eastern fluctuated from 2016 to 2019, with an overall trend of increasing. This trend was also confirmed by recently published literature (36). The authors considered that the reasonable explanation may be the continuous progress urbanization and the increase in traffic flow (37). Wang et al. (33) stated that new drivers and vehicles showed an increasing trend, which result in a serious traffic safety situation. In addition, self-driving tours have become increasingly popular in the western region in recent years. The risk of accidents owing to the driver's unfamiliarity with road conditions was also increasing (38).

The HI, CFR, MRP, and MRV, which represents the severity of traffic accidents were analyzed. These four indicators were almost highest in western China, except the CFR from 2016 to 2019. There has been considerable imbalance in economic development between the western and eastern regions of China. This imbalance may lead to a vast difference in traffic safety owing to several factors including distribution of medical resources, road condition, traffic safety awareness, and traffic management ability (39).

To further investigate the cause of the high severity in western China, the average of HI, CFR, MRP, and MRV were compared among four altitude groups. Although there were fluctuations, the average of these four values were higher for cities located above 3,000 meters than that below 500 meters. It can be derived that high altitude is a related risk factor affecting traffic accidents severity due to the higher proportion of mountain roads. Exposure to high altitudes can cause acute altitude sickness and may lead to several complications including high-altitude cerebral edema, high-altitude pulmonary edema, a reduced metabolic body heat plateau, increased evaporation, and severe dehydration (40–42). These in turn make treatment more difficult. Furthermore, a lack of effective and timely rescue may contribute to the high mortality rate at high altitudes. Mishra et al. (43) concluded that traffic accident related deaths could have been avoided if the injured patients would have been transported to the hospital in time. In a study based on data from Xizang, the lack of health care facilities along the road, severe hypoxia, long evacuation times, and poor knowledge on health care among the evacuees were responsible for the high mortality rate of patients with traffic injuries (44). Wang et al. (45) concluded that in China, the absence of standard procedures for the pre-hospital resuscitation phase, lack of training among emergency medical personnel, and inadequate on-site care result in low success rates of pre-hospital resuscitation.

The above findings suggest that there is a difference in the occurrence and severity of traffic accidents between the eastern and western regions of China. The western region poses a greater public health risk. One hundred and seventy-eight cases of traffic accidents with detailed information illustrated that the significate feature were low-grade road, the combined curved and sloped road, and rollover (Table 3). Peng et al. (46) agreed that driving under unfavorable road conditions was more likely to cause serious crashes, as road sections with curve radii < 1,000 meters or deflection angles < 30° were positively associated with crash injury severity. Mountain roads are also more likely to lead to rollover accidents. A previous study stated that more than 50% of traffic fatalities to passenger car and light truck roll-over crashes occurred in a mountainous road in United States (47). Thus, the poor road condition was an important cause of high severity of traffic accidents in western.

**Table 3:** Descriptive statistics in terms of the frequency, percentages, and chi-square analysis results.

**Variable**	**Category**	**Frequency** ***n*** **(%)**		**Chi-square**	***P*-value**
		**Eastern**	**Western**		
Accident time	Day	76 (67.5)	50 (75.8)	0.031	0.860
	Night	36 (32.1)	16 (24.2)		
Vehicle type	Small passenger car	13 (11.6)	14 (21.2)	4.419	0.110
	Medium/Large passenger car	65 (58.0)	39 (59.1)		
	Truck	34 (30.4)	13 (19.7)		
Road grade	Highway	40 (35.7)	8 (12.1)	26.38	0.000^*^
	Level 1	11 (9.8)	2 (3.0)		
	Level 2	32 (28.6)	16 (24.2)		
	Level 3	13 (11.6)	14 (21.2)		
	Level 4	7 (6.3)	7 (10.6)		
	Other	9 (8.0)	19 (28.8)		
Road shape	Flat straight	58 (51.8)	20 (30.3)	9.574	0.023^*^
	Curve	7 (6.3)	5 (7.6)		
	Slope	16 (14.3)	9 (13.6)		
	Combined curve and slope	31 (27.7)	32 (48.5)		
Accident pattern	Rollover	50 (42.0)	51 (73.9)	21.919	0.001^*^
	Frontal	30 (25.2)	6 (8.7)		
	Rear-end	16 (13.5)	5 (7.3)		
	Fire and other	13 (10.9)	3 (4.4)		
	Side	8 (6.7)	1 (1.4)		
	Fixed object	2 (1.7)	3 (4.3)		
Accident reason	Over speed	60 (21.6)	32 (21.3)	7.178	0.619
	Overload	49 (17.6)	27 (18.0)		
	Bad weather	42 (15.1)	19 (12.7)		
	Vehicle failure	42 (15.1)	25 (16.7)		
	Driver error	29 (10.4)	25 (16.7)		
	Drive opposite	21 (7.6)	7 (4.7)		
	Illegal drive	12 (4.3)	8 (5.3)		
	Fatigue	9 (3.2)	3 (2.0)		
	Illegal passing	8 (2.9)	3 (2.0)		
	Tailgating	6 (2.2)	1 (0.7)		

* Refer to the differences are statistical significance with *p* < 0.05.

In terms human factors contributing to traffic accidents, speeding was the most common behavior. Although there was no statistically significant difference between the western and the eastern. With the implementation of speed limit laws and procedures in China from 2004, fatalities attributed to speeding had been largely reduced (48). Previous study has shown that a lower incidence of traffic accidents in clustered camera areas, which may be a practical key to the effective control of speeding (49). In addition to human factors, we found that the proportion of vehicle failures, such as brake failure, was also high in western regions. Similar studies have shown that driving in hilly terrain requires high vehicle performance. The vehicle breakdown is very likely to lead to traffic accidents (50). Therefore, the driver should check the safety performance of the vehicle in time when driving in the western region.

According to the aforementioned results, there are many aspects of countermeasures to apply, including improving the road safety facilities, restriction of speed, and enhancing medical treatment capabilities at accident scenes. Each Countermeasure will be discussed in detail as follows.

First, improving the road safety facilities. Road conditions are an important factor affecting the occurrence of traffic accidents. Roads at high altitude regions are usually considered to be bad due to topographical features. On the one hand, the government should increase the investment in road construction in the western, especially high-grade roads. On the other hand, safety appurtenances such as guardrails, safety warning signs, and hazard avoidance lanes should be set up at risk roads.

Second, restriction of speed. The government can consider more detailed when formulating speed limit standards, rather than simply determine the speed limit according to the road grade. In addition, operating vehicles can be installed with vehicle operation monitoring system, so that speeding can be detected timely. Fixed speed cameras should also be installed on high accident risk roads. Furthermore, Road safety communication campaigns should be organized frequently, which had been confirmed that the effectiveness of communication campaigns was substantially increased if they are accompanied by other preventive measures such as legislation (51).

Third, enhancing medical treatment capabilities at accident scenes. An authoritative research states that emergency medical service workers are often poorly trained in China, and care received at the site of accidents is often substandard, resulting in a low rate of successful pre-hospital resuscitation (4). Regional differences and inequalities further exacerbate casualties in western (52). How to promote the balanced development of medical and health services should be considered by government authorities. In addition, strengthening the driver's self-help ability is also an essential measure. Consideration should be given to training drivers in basic medical knowledge and installing first aid kits in the vehicle.

### Limitations

There were several limitations to this study. Firstly, the data collected by the police at the scene may have been biased. The fatalities reported by police might be underestimated compare to the data reported by health department (53). Secondly, the hospital records lacked data on the treatment procedures for the injured, which are meaningful for cases of rescue. Furthermore, Multivariate analysis method was not applied in this study due to the small sample size. Maybe it can be completed when more data is available in the future.

## Conclusion

This retrospective analysis of road traffic accidents in western China between 2009 and 2019 showed some distinguish difference of road traffic accidents in the western region existed. The incidence of traffic accidents is decreasing at low altitudes. On the contrary, that in high altitudes is increasing. The severity of traffic accidents in high altitude areas was also found to be higher than that in low altitude areas. This may be related to poorer road conditions and insufficient medical infrastructure. Based on these findings, we further analyzed the characteristics of serious traffic accidents and found differences in road grade, road shape, and accident pattern between the western and eastern regions. Low-grade roads, combined curved and sloping roads, and falling from height were significant features associated with traffic accidents in the western region. Over speeding, overloading, vehicle failure, and bad road conditions were the main factors leading to traffic accidents in the western region. Despite the limitations in this study, the findings suggest that some special measurements should be taken to reduce the crash incidence and their causalities in western regions, especially in high-altitude areas.

## Data availability statement

The original contributions presented in the study are included in the article/Supplementary material, further inquiries can be directed to the corresponding authors.

## Author contributions

JQ, HZ, and MQ were involved in conception and design of the study. GL was involved in data collation. AY and KL were involved in data statistical analysis and interpretation. JQ prepared the draft that was critically reviewed and approved by all the authors. The manuscript has been read and approved by all the authors, and the requirement for authorship was fulfilled by all authors.

## Funding

This research was supported by the National Social Science Foundation of China (2021-SKJJ-C-012), National Nature Science Foundation of China (51975041), Chongqing Postgraduate Research Innovation Project (CYB22286), and the Creative Talents Support Project of Army Special Medical Center (2019CXJSB002).

## Conflict of interest

The authors declare that the research was conducted in the absence of any commercial or financial relationships that could be construed as a potential conflict of interest.

## Publisher's note

All claims expressed in this article are solely those of the authors and do not necessarily represent those of their affiliated organizations, or those of the publisher, the editors and the reviewers. Any product that may be evaluated in this article, or claim that may be made by its manufacturer, is not guaranteed or endorsed by the publisher.

## References

[1] Zou X Vu HL Huang H. Fifty years of accident analysis & prevention: a bibliometric and scientometric overview. Accid Anal Prev. (2020) 144:105568. 10.1016/j.aap.2020.105568 32562929

[2] Maduakonam DE Miriam DU Arthur N. Retrospections on road traffic injuries as a social burden: the role of public health education initiatives in a developing country. Niger J Med. (2015) 24:169–74. 10.4103/1115-2613.278305 26353429

[3] Goniewicz K Goniewicz M Pawłowski W. Road accident rates: strategies and programmes for improving road traffic safety. Eur J Trauma Emerg Surg. (2016) 42:433–8. 10.1007/s00068-015-0544-6 26162937

[4] Jiang B Liang S Peng ZR. Transport and public health in China: the road to a healthy future. Lancet. (2017) 390:1781–91. 10.1016/S0140-6736(17)31958-X 29047445 PMC5704968

[5] Zhao H Yin Z Xiang H. Preliminary study on alterations of altitude road traffic in China from 2006 to 2013. PLoS ONE. (2017) 12:e0171090. 10.1371/journal.pone.0171090 28187203 PMC5302387

[6] Cheng S Xiang Z Xi H. Environmental status and human health: evidence from China. Int J Environ Res Public Health. (2022) 19:12623. 10.3390/ijerph191912623 36231923 PMC9566106

[7] Wang F Chen H Zhu CH. Estimating driving fatigue at a Plateau area with frequent and rapid altitude change. Sensors. (2019) 19:4982. 10.3390/s19224982 31731740 PMC6891775

[8] Yuan Z Huang D Tong W. Characteristic analysis and prediction of traffic accidents in the multiethnic Plateau mountain area. J Transport Eng Part a-Systems. (2020) 146:04020068. 10.1061/JTEPBS.0000398

[9] Yang Y Peng Y He S. The clinical differences of patients with traumatic brain injury in Plateau and Plain Areas. Front Neurol. (2022) 13:848944. 10.3389/fneur.2022.848944 35547378 PMC9081812

[10] Luo W Xie Y. Economic growth, income inequality and life expectancy in China. Soc Sci Med. (2020) 256:113046. 10.1016/j.socscimed.2020.113046 32446156

[11] Liu G Chen S Zeng Z. Risk factors for extremely serious road accidents: results from national road accident statistical annual report of China. PLoS ONE. (2018) 13:e0201587. 10.1371/journal.pone.0201587 30067799 PMC6070265

[12] Sun LL Liu D Chen T. Road traffic safety: an analysis of the cross-effects of economic, road and population factors. Chin J Traumatol. (2019) 22:290–5. 10.1016/j.cjtee.2019.07.004 31506232 PMC6823720

[13] Wegman F Allsop R Antoniou C. How did the economic recession (2008-2010) influence traffic fatalities in OECD-countries? Accid Anal Prev. (2017) 102:51–9. 10.1016/j.aap.2017.01.022 28259828

[14] Pei YY Wang XS Yang MM. Comparative analysis of traffic safety characteristics of typical metropolis in China. Auto Safety. (2021) 1:1–7. 10.3969/j.issn.1006-6713.2021.09.021

[15] Hayakawa H Fischbeck PS Fischhoff B. Traffic accident statistics and risk perceptions in Japan and the United States. Accid Anal Prev. (2000) 32:827–35. 10.1016/S0001-4575(00)00007-5 10994610

[16] Wang D Liu Q Ma L. Road traffic accident severity analysis: census-based study in China. J Safety Res. (2019) 70:135–47. 10.1016/j.jsr.2019.06.002 31847989

[17] Ng KS Hung WT Wong WG. An algorithm for assessing the risk of traffic accident. J Safety Res. (2002) 33:387–410. 10.1016/S0022-4375(02)00033-6 12405000

[18] Ball K Edwards JD Ross LA. Cognitive training decreases motor vehicle collision involvement of older drivers. J Am Geriatr Soc. (2010) 58:2107–13. 10.1111/j.1532-5415.2010.03138.x 21054291 PMC3057872

[19] Pakgohar A Tabrizi RS Khalili M. The role of human factor in incidence and severity of road crashes based on the CART and LR regression: a data mining approach. Procedia Comput Sci. (2011) 3:764–9. 10.1016/j.procs.2010.12.126

[20] Zhang Y Jing L Sun C. Human factors related to major road traffic accidents in China. Traffic Inj Prev. (2019) 20:796–800. 10.1080/15389588.2019.1670817 31710507

[21] Xing Y Chen S Zhu S. Exploring risk factors contributing to the severity of hazardous material transportation accidents in China. Int J Environ Res Public Health. (2020) 17:1344. 10.3390/ijerph17041344 32093095 PMC7068398

[22] Jamal A Umer W. Exploring the injury severity risk factors in fatal crashes with neural network. Int J Environ Res Public Health. (2020) 17:7466. 10.3390/ijerph17207466 33066522 PMC7602238

[23] Martins MA Garcez TV. A multidimensional and multi-period analysis of safety on roads. Accid Anal Prev. (2021) 162:106401. 10.1016/j.aap.2021.106401 34562683

[24] Wang X Qu Z Song X. Incorporating accident liability into crash risk analysis: a multidimensional risk source approach. Accid Anal Prev. (2021) 153:106035. 10.1016/j.aap.2021.106035 33607319

[25] Traffic Management Bureau of the Public Security Ministry of the People's Republic of China. Road Traffic Accident Annual Census Report of China. Beijing: Traffic Management Bureau (2009–2019).

[26] Gansu Statistics Bureau. Shanghai Statistical Yearbook (2009–2019).

[27] Tibet Statistics Bureau. Tibet Statistical Yearbook (2009–2019).

[28] Guizhou Statistics Bureau. Guizhou Statistical Yearbook (2009–2019).

[29] Qinghai Statistics Bureau. Qinghai Statistical Yearbook (2009–2019).

[30] Sichuan Statistics Bureau. Sichuan Statistical Yearbook (2009–2019).

[31] Xinjiang Statistics Bureau. Xinjiang Statistical Yearbook (2009–2019).

[32] Yunnan Statistics Bureau. Yunnan Statistical Yearbook (2009–2019).

[33] Wang X Yu H Nie C. Road traffic injuries in China from 2007 to 2016: the epidemiological characteristics, trends and influencing factors. PeerJ. (2019) 7:e7423. 10.7717/peerj.7423 31404405 PMC6688591

[34] Sun LL Liu D Chen T. Analysis on the accident casualties influenced by several economic factors based on the traffic-related data in China from 2004 to 2016. Chin J Traumatol. (2019) 22:75–9. 10.1016/j.cjtee.2019.02.002 30962129 PMC6488516

[35] Vecino-Ortiz AI Nagarajan M Elaraby S. Saving lives through road safety risk factor interventions: global and national estimates. Lancet. (2022) 400:237–50. 10.1016/S0140-6736(22)00918-7 35779550

[36] Qi M Hu X Li X. Analysis of road traffic injuries and casualties in China: a ten-year nationwide longitudinal study. PeerJ. (2022) 10:e14046. 10.7717/peerj.14046 36128192 PMC9482767

[37] Mao X Yuan C Gan J. Risk factors affecting traffic accidents at urban weaving sections: evidence from China. Int J Environ Res Public Health. (2019) 16:1542. 10.3390/ijerph16091542 31052370 PMC6539961

[38] Wang X Wang M Lu X. Spatio-temporal evolution and driving factors of the high-quality development of provincial tourism in China. Chin Geogr Sci. (2022) 32:896–914. 10.1007/s11769-022-1307-z 36091643 PMC9446628

[39] Leisman G Waksman I. Commentary: The incidence of road traffic crashes among young people aged 15-20 years: differences in behavior, lifestyle and sociodemographic indices in the Galilee and the Golan. Front Public Health. (2021) 9:651376. 10.3389/fpubh.2021.651376 33748072 PMC7969495

[40] Burtscher M Gatterer H Burtscher J. Extreme terrestrial environments: life in thermal stress and hypoxia. A narrative review. Front Physiol. (2018) 9:572. 10.3389/fphys.2018.00572 29867589 PMC5964295

[41] Gatterer H Wille M Faulhaber M. Association between body water status and acute mountain sickness. PLoS ONE. (2013) 8:e73185. 10.1371/journal.pone.0073185 24013267 PMC3754926

[42] Wang H Zhu X Xiang H. Effects of altitude changes on mild-to-moderate closed-head injury in rats following acute high-altitude exposure. Exp Ther Med. (2019) 17:847–56. 10.3892/etm.2018.7020 30651871 PMC6307396

[43] Mishra B Sinha Mishra ND Sukhla S. Epidemiological study of road traffic accident cases from Western Nepal. Indian J Community Med. (2010) 35:115–21. 10.4103/0970-0218.62568 20606934 PMC2888338

[44] Wang Q Yu X Hu X. Analysis on 1,894 cases of road traffic injuries in the Qinghai-Tibet Plateau. Chin J Trauma. (2004) 20:136–8. 10.3760/j:issn:1001-8050.2004.03.003

[45] Wang T Yin X Zhang P. Road traffic injury and rescue system in China. Lancet. (2015) 385:1622. 10.1016/S0140-6736(15)60794-2 25943819

[46] Peng Z Wang Y Wang L. A comparative analysis of factors influencing the injury severity of daytime and nighttime crashes on a mountainous expressway in China. Int J Inj Contr Saf Promot. (2021) 28:503–12. 10.1080/17457300.2021.1964089 34392808

[47] Alrejjal A Farid A Ksaibati K. Investigating factors influencing rollover crash risk on mountainous interstates. J Safety Res. (2022) 80:391–8. 10.1016/j.jsr.2021.12.020 35249620

[48] He J King M Watson B. Speed enforcement in China: national, provincial and city initiatives and their success. Accid Anal Prev. (2013) 50:282–8. 10.1016/j.aap.2012.04.017 22579218

[49] Malekpour MR Azadnajafabad S Rezazadeh-Khadem S. The effectiveness of fixed speed cameras on Iranian taxi drivers: an evaluation of the influential factors. Front Public Health. (2022) 10:964214. 10.3389/fpubh.2022.964214 36111189 PMC9468364

[50] Lu H ji X Yang W. Cause analysis of different patterns of traffic accidents on plateau mountain roads. China Safety Sci J. 29:44–9.

[51] Faus M Alonso F Fernández C. Are traffic announcements really effective? A systematic review of evaluations of crash-prevention communication campaigns. Safety. (2021) 7:66. 10.3390/safety7040066

[52] Chen B Jin F. Spatial distribution, regional differences, and dynamic evolution of the medical and health services supply in China. Front Public Health. (2022) 10:1020402. 10.3389/fpubh.2022.1020402 36211684 PMC9540227

[53] Huang H Yin Q Schwebel DC. Examining road traffic mortality status in China: a simulation study. PloS ONE. (2016) 11:e0153251. 10.1371/journal.pone.0153251 27071008 PMC4829231

